# Altered gut microbiome promotes proteinuria in mice induced by Adriamycin

**DOI:** 10.1186/s13568-018-0558-7

**Published:** 2018-02-28

**Authors:** Qian Jiang, Xiwei He, Yuntao Zou, Yin Ding, Huang Li, Huimei Chen

**Affiliations:** 1 Orthodontic Department, Nanjing Stomatological Hospital, Medical School of Nanjing University, Nanjing, Jiangsu China; 2 State Key Laboratory of Pollution Control and Resource Reuse, School of the Environment, Nanjing University, Nanjing, Jiangsu China; 3 Institute of Medical Genetics, Medical School of Nanjing University, Nanjing, Jiangsu China; 4 Jiangsu Key Laboratory of Molecular Medicine, Department of Medical Genetics, Nanjing University, Nanjing, Jiangsu China

**Keywords:** Gut microbiome, Proteinuria, Chronic kidney disease, Adriamycin

## Abstract

Inflammation has recently been attributed to dysbiosis of the gut microbiome, which has been linked to proteinuria in chronic kidney disease. Since Adriamycin^®^ (ADR) is widely used to induce proteinuria in mouse models, the aim of this study was to explore the potential effect of gut microbiome on this process. Both ADR resistant (C57BL/6) and susceptible (BALB/C) strains were part of the induced nephropathy with ADR injection. BALB/C mice significantly presented increased urinary albumin/creatinine ratio (UACR) with renal lesions in pathology, but C57BL/6 mice were absent from kidney damage. Species and genus level resolution analysis showed a shift in gut microbial profile between BALB/C and C57BL/6 mice. ADR further altered the stool microbiome in BALB/C mice, particularly with enrichment of *Odoribacter* and depletion of *Turicibacter*, *Marvinbryantia* and *Rikenella*. Moreover, the level of UACR in BALB/C mice was marked related to the abundance of *Marvinbryantia*, *Odoribacter* and *Turicibacter* in stool. Meanwhile, ADR remarkably increased the serum levels of interleukin (IL)-2 in BALB/C mice, but not in C57BL/6 mice. It is suggested that the favorably altered stools as shown in the microbiome might promote the inflammation and proteinuria in ADR-sensitive mice, which provides a new insight on the pathogenicity of chronic kidney disease.

## Introduction

Chronic kidney disease (CKD) is a major public health problem that has received increasing attention because of its association with the high rate of end stage renal disease (ESRD) (Eknoyan et al. 2004; Liu et al. 2015; Martinez-Castelao et al. 2014). Proteinuria is often observed in patients with CKD, and it is reportedly a vital risk factor for the progression and prognosis of CKD (Sandsmark et al. 2015). Infections, toxins, diabetes and high blood pressure have been investigated in the pathogenesis of proteinuria and glomerulosclerosis. The status of inflammation is also suggested to be involved in proteinuria development. However, the source and alternation of inflammation in CKD has been ignored, especially in those patients without infection, auto-immunology disease and allergic disease.

Recently, an increased interest in the role of gut microbiome has gained momentum and increasing evidence supports a kidney-gut axis (Evenepoel et al. 2016; Vaziri et al. 2013). The dysbiosis of the gut microbiota was observed in CKD, which produces key neurovascular toxins, including indoxyl sulphate (IS) and *p*-cresyl sulphate (PCS) (Evenepoel et al. 2016; Okuda et al. 1986). Moreover, these toxins and their associations would increase inflammation, kidney disease progression and then cause mortality in the CKD population (Evenepoel et al. 2016; Stenvinkel 2005; Vaziri et al. 2013). There is a need for conclusive findings that can elucidate whether there is a causal role of gut microbiome in proteinuria.

To test the hypothesis that the dysbiosis in the gut microbiome accompanied by inflammation can cause proteinuria, we analyzed the gut microbiome profile in the Adriamycin (ADR)-induced nephropathy in BALB/C mice, which was an ADR susceptible strain, as well C57BL/6, an ADR resistant strain that served as a control. The correlations of significantly altered microbes with urine protein levels and the levels of inflammation were demonstrated. It suggested an altered stool microbiome might promote the inflammation and proteinuria in ADR-sensitive mice.

## Materials and methods

### Experimental animals

6-week-old mice with inbred strains of BALB/C and C57BL/6 were obtained from the experimental animal center of the Academy of Military Medical Science of China. All animal study protocols were approved by the Vanderbilt University Institutional Animal Care and Use Committee. Male BALB/C mice were divided into two groups with six animals in each, so were C57BL/6. Mice were housed in a specific pathogen free environment with temperature- and humidity-controlled environment under 12 h light/12 h dark conditions. They were fed a standard rodent diet in accordance with our institutional guidelines for the care and use of laboratory animals.

To establish the animal model of ADR-induced nephropathy, BALB/C and C57BL/6 mice received a single intravenous injection of ADR (10.0 mg/kg; Sigma, St. Louis, MO). Control mice were treated with an equivalent intravenous volume of normal saline (NS). The urinary samples were individually collected using metabolism cages at 0 h, 1 and 2 weeks, and the mice were killed at 2 weeks after ADR or NS injection. Cardiac blood, urine, stool and kidney tissue were collected for analysis. Nine C57BL/6 and BALB/C male mice were used in each group. ADR injection duration was varied from 24 h to 7 weeks with an optimal dose of 10–11 mg/kg body wt of ADR (Sun et al. 2013; Wang et al. 2000). In BALB/C mice, glomerular endothelial cells undergoing apoptosis could be detected as early as 24 h after ADR administration (Sun et al. 2013). While the overt proteinuria was reported to appear at day 5 and was maximal at day 7 (Wang et al. 2000). Therefore, we chose 1 and 2 weeks after ADR treatment for blood, urine and kidney tissue collection.

### Measurements of serum or urine parameters

The serum was separated by allowing the whole blood to stand at room temperature for 2 h. Creatinine concentrations were measured using a Creatinine (CREA) reagent (Shanghai Gaozong Medical Technology Co, Shanghai, China) and urine albumin concentrations were determined using the BCA Protein Assay Kit (Thermo Scientific, Waltham, MA, USA). CCr (creatinine clearance) was calculated according to a Cockcroft–Gault formula: urinary volume (ml) × urinary creatinine (mg/dl))/(plasma creatinine (mg/dl) × urine collection length (min) (Hsu et al. 2016). Results were expressed as ml/min/100 g body weight. The urinary albumin-to-creatinine ratio (UACR) in g/mol was calculated as the urinary albumin concentration divided by the creatinine concentration.

Interleukin-2 (IL-2) levels in serum were determined by ELISA. ELISA assays were carried out using an immunoassay kit—Mouse IL-2 Quantikine ELISA Kit M2000, (R&D Systems, Minneapolis, MN) according to the manufacturer’s protocol.

### Histological analysis of kidney

Mouse kidney samples were fixed in 10% formaldehyde, embedded in paraffin and cut into 4 μm sections. Each section was routinely stained with H&E. The pathological changes were observed using a light microscope and photographs were obtained. Sections were examined by a qualified pathologist who was blinded to other data. 10 images were captured and scored for each sample. The pathologist’s job was to evaluate the degree of pathological changes based on glomerular epithelial hyperplasia.

### High-throughput sequencing of fecal DNA

Genomic DNA from fecal samples (50–100 mg) was extracted using a FastDNA SPIN kit for soil (MP Biomedicals, CA, USA). The concentrations and purity of the resultant DNA were determined by a NanoDrop (NanoDrop ND-2000, USA) and stored at − 80 °C for further analysis.

The 16S rRNA gene in the fecal DNA samples was amplified by polymerase chain reaction (PCR) with primers 16s-F (5′-AGAGTTTGATYMTGGCTCAG-3′) and 16s-R (5′-TGCTGCCTCCCG TAGGAGT-3′) targeting the hypervariable V1–V2 region of the 16S rRNA gene of bacteria. PCR products were purified using a DNA Fragment Purification Kit (Takara, Japan), and then barcoded and pooled to construct the sequencing library. An Illumina Mi-seq (Illumina, USA) was used for sequencing to generate 150,150 reads. The sequences obtained were deposited in a NCBI Sequence Read Archive under accession number PRJNA327732.

### Post-run analysis

After sequencing, the raw data was sorted into different samples according to the barcodes. A sickle tool was applied to perform quality filtering of the raw reads and the quality was greater than 20 without any unknown bases (Joshi and Fass 2011). The modified pipeline (http://www.mothur.org/wiki/MiSeq_SOP) was used for the bioinformatics analysis and the number of the sequences was normalized by randomly extracting 29,966 reads to fairly compare all the samples at the same sequencing depth.

‘Aligner’ and ‘Complete Linkage Clustering’ were applied to calculate the operational taxonomic units (OTUs) with a threshold of 97% sequence similarity. The Chao index was then calculated using Mothur. Taxonomic classification of each sample was individually conducted using Ribosomal Database Project (RDP) Classifier (version 2.6) with a confidence threshold of 80%. Cluster analysis was conducted with paleontological statistics software (PAST, version 3.01). The heat map of bacteria genus was conducted with R Language (version 3.1).

### Statistical analysis

All data were presented as mean ± SEM and analyzed using SPSS version 20.0. Comparisons were performed using an independent *t* test for two groups or ANOVA (one-way analysis of variance) for four groups when parameters showed a normal distribution; otherwise it was performed using non-parametric tests. Repeated-measures ANOVA was used to test the difference of weight, CCr and UACR for different groups at three time points. The sequence number data of gut microbiome were normalized by log10 transformations prior to correlation analysis. The association between proteinuria and gut microbiome was revealed by bivariate correlation analysis and logistic regression analysis. Differences were considered significant at *p* < 0.05.

## Results

### BALB/C mice were sensitive to ADR

**Fig. 1:**
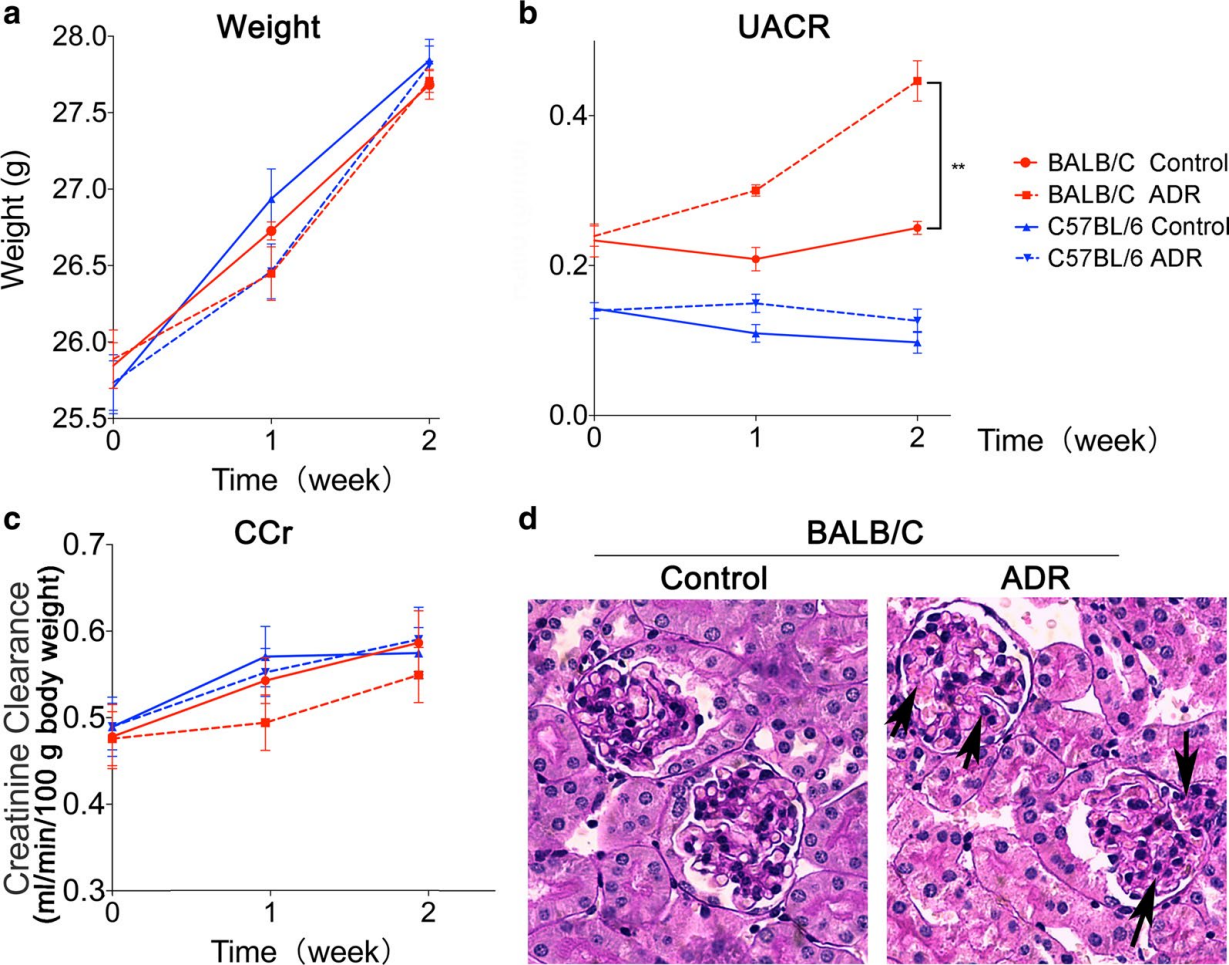
BALB/C mice were sensitive to ADR. **a** The weight changes in the control and ADR group of different mice during the 2 weeks; **b** the UACR changes in the control and ADR group of different mice during the 2 weeks; **c** the CCr changes in the control and ADR group of different mice during the 2 weeks; **d** the nephridial tissue section of BALB/C ADR group and control group, glomeruli damage is indicated by filled arrows. **p < 0.01

Before ADR injection, BALB/C mice presented similar levels of body weight (p = 0.543) and CCr (p = 0.824), but higher levels of UACR (p = 0.003) when compared with C57BL/6 mice (Fig. 1a–c). The ADR injection further enhanced the difference of UACR between BALB/C and C57BL/6 mice (p < 0.001). After ADR injection, the UACR levels were significantly increased by twofold (p < 0.001) in BALB/C mice. ADR also lead to histological lesions in BALB/C mice. Figure 1d shows mesangial proliferation and segmental glomerular sclerosis in glomeruli in the BALB/C ADR group. However, ADR had no impact on C57BL/6 mice at body weight, CCr and UACR.

### Gut microbiome altered with ADR in BALB/C mice

**Fig. 2:**
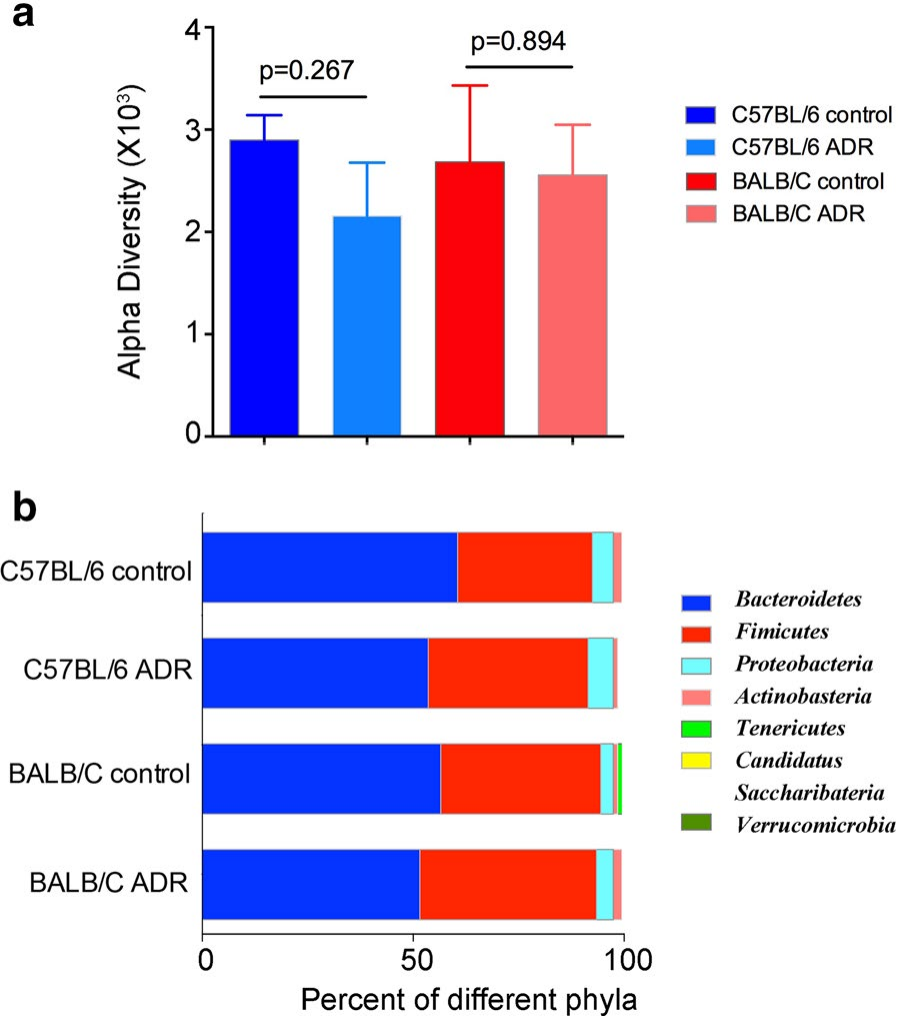
Gut microbiome altered with ADR at phylum level. **a** The alpha diversity in the control and ADR group of different mice. **b** The gut microbiome composition profiles at the phylum level

Gut microbiome were determined by Illumina Mi-seq sequencing using stool samples. Alpha diversity in BALB/C and C57BL/6 mice was 2683.54 ± 492 and 2897.85 ± 244, respectively (p = 0.75, Fig. 2a). A slight decrease of microbiome diversity was observed after ADR exposure, but it did not reach the statistical significance (both p > 0.05). At phylum level, *Bacteroidetes* (52–61%) and *Firmicutes* (32–42%) were predominant and followed by *Proteobacteria* (3–6%), *Actinobacteria* (1–2%) and *Tenericutes* (0–2%) (Fig. 2b). There was no significant difference between the two kinds of mice, and ADR injection did not obviously alter the phylum distribution, either.

**Fig. 3:**
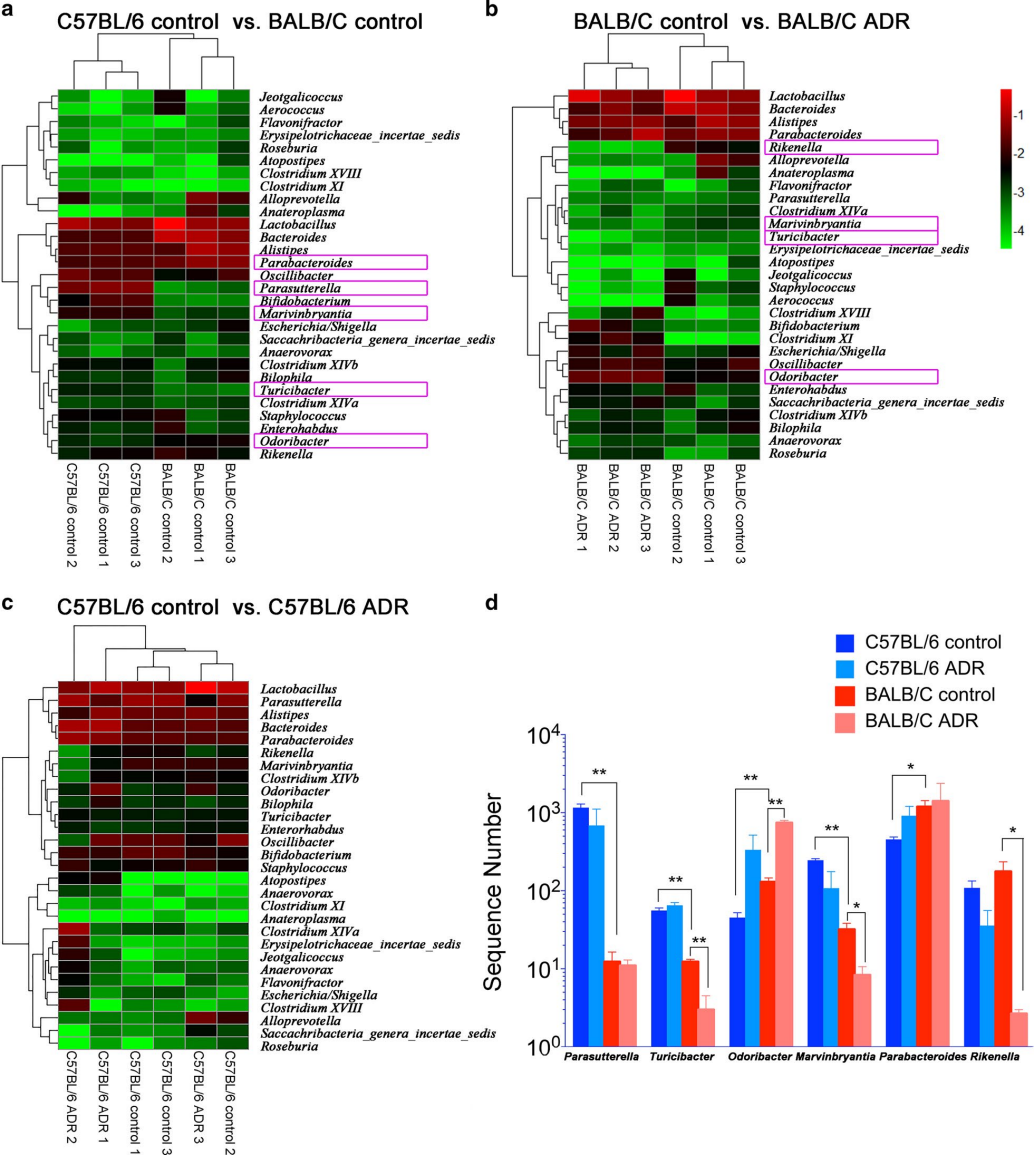
Gut microbiome altered with ADR at genus level. **a** The gut microbiome composition of C57BL/6 control group and BALB/C control group; **b** the gut microbiome composition of BALB/C ADR group and BALB/C control group; **c** the gut microbiome composition of C57BL/6 ADR group and C57BL/6 control group; **d** certain genera that were significantly different between BALB/C and C57BL/6 mice or had changed after ADR treatment. *p < 0.05; **p < 0.01

Figure 3 shows an interesting separation of the top 1% genera between BALB/C and C57BL/6 mice. Compared with C57BL/6 mice, BALB/C presented higher abundance of *Parabacteroides* in *Bacteroidetes* (p = 0.038) and *Odoribacter* in *Bacteroidetes* (p = 0.008), but a lower abundance of *Parasutterella* in *Proteobacteria* (p = 0.015), *Marvinbryantia* in *Firmicutes* (p < 0.001) and *Turicibacter* in *Firmicutes* (p = 0.001). The copy number for three of these five genera were further altered by ADR treatment in BALB/C mice, but was similar in C57BL/6 mice before and after treatment. In BALB/C mice, *Turicibacter* was significantly decreased by 75.7% (p = 0.006) and *Marvinbryantia* by 74.0% (p = 0.024), while *Odoribacter* was increased around fourfold (p < 0.001). Besides, *Rikenella* was also significantly decreased by 98.5% (p = 0.041).

### Altered gut microbiome associated with proteinuria

**Fig. 4:**
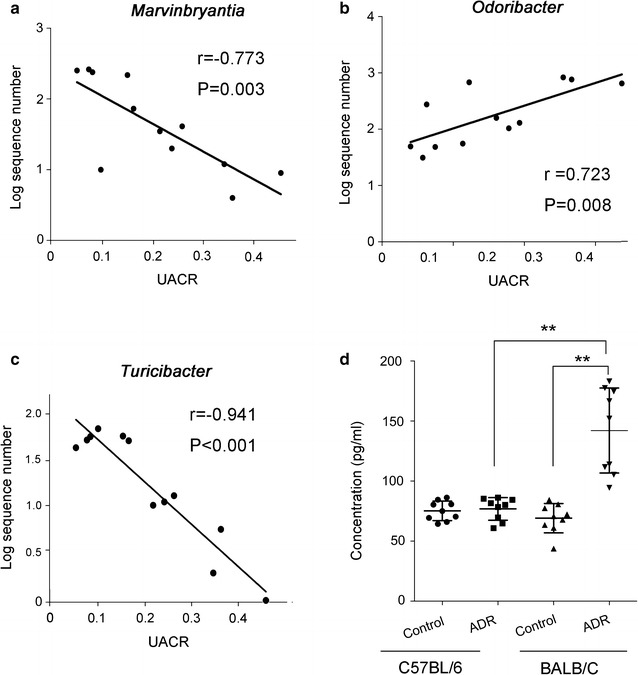
The association between the microbiomes and the UACR. **a** The association between the *Marvinbryantia* and the UACR. **b** The association between the *Odoribacter* and the UACR. **c** The association between the *Turicibacter* and the UACR. **d** The serum IL-2 concentrations of the four groups

Since ADR led to the distinct gut microbiome alternation in BALB/C mice with renal damage, an association between increased proteinuria and altered gut microbiome was proposed as viable. We then analyzed three genera, which were different between two kinds of mice and changed after ADR treatment in BALB/C mice (Fig. 4). Results showed that *Marvinbryantia* (r = − 0.773, p = 0.003), *Odoribacter* (r = 0.723, p = 0.008) and *Turicibacter* (r = 0.941, p < 0.001) were directly associated with the levels of UACR. Multiple regression analysis about the three microbiome and UACR revealed that *Turicibacter* is independently associated with the levels of UACR (R^2^ = 0.898, p < 0.001).

Furthermore, we detected the serum IL-2 concentration in BALB/C and C57BL/6 mice (Fig. 4d). Two strains presented similar serum levels of IL-2 at baseline (p = 0.481). ADR exposure obviously increased the levels of IL-2 in BALB/C mice (p < 0.001), but not in C57BL/6 mice (p = 0.623). These data suggested that increased IL-2 levels might be involved in the process of ADR treatment in BALB/C mice, while the stable levels of IL-2 in C57BL/6 mice were parallel to their resistance to ADR.

## Discussion

In the present study, we confirmed that BALB/C mice are sensitive to ADR-induced nephropathy, while C57BL/6 mice are resistant to such damage. We also showed the distinct gut microbiome and its association with renal damage between BALB/C and C57BL/6 mice. IL-2 was further indicated as being involved in the process of ADR treatment, although the underlying mechanism is far from clear. To the best of our knowledge, this study is the first report indicating the contribution of certain altered gut genus for the increased UACR in an animal model.

Bertani et al. (1982) reported that ADR can induce nephrotic syndrome with morphologic changes in the kidney. Jeansson et al. (2009) suggested that ADR induces direct cell toxic damage to the glomerulus with subsequent tubulointerstitial injury. Lee and Harris (2011) further showed that the early nephrotoxic effect of ADR was related to a generation of oxygen free radicals. Since ADR-induced nephropathy mimicking human proteinuric disease, it is widely used in the study of pathogenesis of renal damage as well as in pharmacology (Cheng et al. 2007; Guo et al. 2008; Wu et al. 2014). However, ADR-induced nephropathy has been found to be species dependent. C57BL/6 mice are normally an ADR resistant strain, while BALB/C mice are an ADR susceptible strain (Sun et al. 2013). We confirmed this species difference in the present study. Until now, the underlying mechanism have not been substantiated, but we have provided a hypothesis about the possible mechanism for the different reaction between these 2 species of mice. For BALB/C mice, ADR treatment influenced the diversity and function of the gut microbiota with implications for health. Disturbance of the gut flora, referred to as ‘gut dysbiosis’, (i.e., the reduction of Turicibacter and Marvinbryantia, and the increase of Odoribacter) induced a disruption of the epithelial barrier, ultimately resulting in an increased serum IL-2 level. The renal toxicity came from the metabolites and an association were found between the gut microbiome alteration and the urinary albumin/creatinine ratio (UACR). While for ADR resistant C57BL/6 mice, there were no significant alteration of the gut microbiota after ADR treatment. Unaffected gut microbiota may avoid a disruption of the epithelial barrier and following disease. To support this hypothesis about the mechanism, future researches should demonstrated that restoration of gut microbial composition in BALB/C mice treated with ADR (e.g., by means of probiotic or fecal transplant therapy) would improve albuminuria and/or inflammation.

In this study explored the difference of gut microbiome composition and reaction to the ADR treatment, and found that it might prompt the distinct renal response. C57BL/6 mice demonstrated a distinct profile of microbiome file in gut. For BALB/C mice, ADR induced obvious proteinuria with a significant change in gut microbiome, and their copy number was directly associated with proteinuria. However, C57BL/6 mice were resistant to ADR treatment and absent from kidney damage and gut microbiome alteration. ADR, as an anthracycline antibiotic, is widely used in solid and hematopoietic malignancy therapies. It is considered as one of the simplest and most effective chemotherapeutic agents (Liu et al. 2013), while several significant side effects are also reported, such as ADR induced cardiomyopathy, typhlitis and proteinuria (Chen et al. 2015; Pestalozzi et al. 1993). ADR has also been known to induce gastrointestinal epithelial damage (Fahim et al. 2011) and alter the composition of gut microbiota, which induces the translocation of bacteria species and then stimulates immune responses (Viaud et al. 2013). These finding support the association between gut microbiome and proteinuria under ADR treatment in certain species.

Gut microbiome phyla composition was similar among different mice, which is also in consistent with previous reports (Sommer and Backhed 2013). The ADR treatments led to a decreased pattern of alpha diversity, suggesting a light poison exposure (Zhang et al. 2015). In the model of BALB/C mice, we illustrated the significance of *Marvinbryantia*, *Odoribacter* and *Turicibacter* and highlighted the independent effect of *Turicibacter* associated with UACR.

*Turicibacter* was reported to be involved in anti-inflammatory properties (Presley et al. 2010; Suchodolski et al. 2012). It has been verified that the levels of *Turicibacter* decrease with idiopathic inflammatory bowel diseases (IBD) in dog models (Suchodolski et al. 2012); while they increase in colitis-resistant mice when compared to those sensitive to IBD (Presley et al. 2010). In this study, we demonstrated that *Turicibacter* was at a lower abundance in BALB/C mice than in C57BL/6 mice and it even decreases after ADR treatment. We also showed a significant negative correlation between *Turicibacter* and proteinuria. Considering the relationship between inflammation and kidney damage, our data supported the possible anti-inflammatory effect of *Turicibacter* in the mice model of mice treated with ADR. The significance of *Odoribacter* and *Marvinbryantia* was also demonstrated in this model.

*Odoribacter* was described to contribute to the inflammatory process and be involved in IBD, Crohn’s disease and ulcerative colitis (Stenvinkel 2005). Our study found that *Odoribacter* was higher in BALB/C mice, and it even increased after ADR treatment, indicating a direct association of *Odoribacter* and proteinuria. Little research has mentioned *Marvinbryantia,* which was lower in BALB/C mice than in C57BL/6 mice and even decreased after ADR treatment in this study.

In addition, cytokine IL-2 is often considered be correlated with gut microbiome level (Bajaj et al. 2012). Certain families, including *Alcaligeneceae*, *Porphyromonadaceae* and *Enterobacteriaceae*, were strongly associated with IL-2 levels in patients with hepatic encephalopathy (Bajaj et al. 2012). Increasing functions of IL-2 have proven to be resistant to microorganism infection and regulation of Tregs growth, survival and activity (Ohkura et al. 2011), which play a role in IBD (Waidmann et al. 2002), cirrhosis (Bajaj et al. 2012), and itchy psoriasis (Reich and Szepietowski 2007). Although the underlying mechanism is not clear, our data showed the association of IL-2 with ADR-induced proteinuria. The serum IL-2 obviously increased with ADR exposure in BALB/C mice. Parallel to the resistance to ADR-induced proteinuria, the serum IL-2 had no significant change after ADR treatment in C57BL/6 mice.

Thus, our findings suggested a new insight to understanding the ADR-induced proteinuria and established the relationship between the gut microbiome and the proteinuria in vivo. Although we could not present a detailed report showing the effect of gut microbiome on the kidney, we did show the difference of response to ARD treatment between BALB/C and C57BL/6 mice and provided a possible mechanism. This research revealed kidney damage, microbiome alteration and a level of inflammation due to ADR treatment. The reason for the different responses of microbiome to ADR treatment is still a mystery. More investigation is also needed to clarify the detail mechanism of increased IL-2 involved in kidney damage and microbiome alteration.

## Abbreviations

ADR: Adriamycin
UACR: urinary albumin/creatinine ratio
IL: levels of interleukin
CKD: chronic kidney disease
ESRD: end stage renal disease
IS: indoxyl sulphate
PCS: *p*-cresyl sulphate
NS: normal saline
CREA: creatinine
CCr: creatinine clearance
PCR: polymerase chain reaction
OTUs: the operational taxonomic units
RDP: Ribosomal Database Project
ANOVA: one-way analysis of variance
IBD: inflammatory bowel diseases
